# Supplying Bee Pollen and Propolis to Growing Rabbits: Effects on Growth Performance, Blood Metabolites, and Meat Quality

**DOI:** 10.3390/life12121987

**Published:** 2022-11-28

**Authors:** María Inés Sierra-Galicia, Raymundo Rodríguez-de Lara, José Felipe Orzuna-Orzuna, Alejandro Lara-Bueno, José Guadalupe García-Muñiz, Marianela Fallas-López, Pedro Abel Hernández-García

**Affiliations:** 1 Posgrado en Producción Animal, Departamento de Zootecnia, Universidad Autónoma Chapingo, Texcoco 56230, Mexico; 2 “Conejos” Centro de Investigación Científica del Estado de México A.C. (COCICEMAC), Coatlinchan 56250, Mexico; 3 Centro Universitario UAEM Amecameca, Universidad Autónoma del Estado de México, Amecameca 56900, Mexico

**Keywords:** honeybee products, coccidiosis, blood biochemistry, hematological profile

## Abstract

The objective of this study was to evaluate the effect of supplementation with bee pollen (BP) and propolis (PRO) on productive performance, *Eimeria* oocyst counts in feces, blood metabolites, and the meat quality of growing rabbits. A total of 160 hybrid rabbits (California × New Zealand) of 30 days of age and 643 ± 8.0 g body weight (BW) were assigned to four treatments with 10 replicates each (four rabbits/replicate). The treatments were as follows: (1) CON: rabbits fed basal diet and not supplemented with BP or PRO; (2) BP500: CON + BP (500 mg/kg BW); (3) PRO50: CON + PRO (50 µL/kg BW); and (4) BP + PRO: CON + BP (500 mg/kg BW) + PRO (50 µL/kg BW). Higher daily weight gain (*p* = 0.04) and lower feed conversion rate (*p* = 0.03) were observed in rabbits supplemented with PRO50. In addition, supplementation with PRO50 and BP + PRO reduced the amount of *Eimeria* oocysts per gram of feces (*p* < 0.05). Most hematological and serum biochemical parameters were similar in rabbits of all treatments. Protein content, collagen, and meat color were similar between treatments. In conclusion, propolis supplementation (50 µL/kg BW) can prevent coccidiosis and act as a natural growth promoter in rabbits without affecting animal health and meat quality.

## 1. Introduction

It has been discussed that, due to its short production cycle, rabbit production could be used as an alternative to solve the growing meat shortage in developing countries [1]. However, due to their complex digestive physiology, rabbits are susceptible to enteric diseases, mainly in the post-weaning period [2]. Therefore, antibiotics (e.g., zinc bacitracin) are frequently used in feeding growing and fattening rabbits to improve productive performance and reduce mortality caused by digestive disorders [3]. Unfortunately, due to the uncontrolled use of antibiotics, a rapid increase in microbial resistance to these drugs has been reported worldwide, compromising the health of humans and animals [4]. Consequently, in recent years the search for and development of new natural products that can improve animal performance and health has attracted the attention of researchers [5]. Some bee (*Apis mellifera)* products, such as bee pollen (BP) and propolis (PRO), could be used for this purpose because they contain various nutritional compounds and some bioactive metabolites with pharmaceutical properties [5,6].

BP is a mixture of flower pollen grains collected by bees with nectar and secretions from the hypopharyngeal glands of bees [6,7]. According to Thakur and Nanda [8], BP contains carbohydrates, proteins, lipids, fiber, minerals, and phenolic compounds (on average 30.59 mg gallic acid/g DM) with anti-inflammatory, antioxidant, and hepatoprotective activity. Some researchers have mentioned that dietary inclusion of BP can be used to improve productive and reproductive performance and intestinal health in domestic animals [6]. Particularly in growing rabbits, supplementation with BP increases nutrient digestibility, volatile fatty acid production in the cecum, and digestive enzyme activity [9]. On the other hand, in growing rabbits, supplementation with BP improves intestinal morphology [10] and increases the serum concentration of insulin-like growth factor type 1 [11]. Previous studies [1,2,11] have evaluated the effects of BP supplementation in rabbits, mainly using low doses (≤350 mg/kg BW); however, information on the effects of high doses (>350 mg/kg BW) of BP is limited. For example, Abdel-Hamid et al. [11] and Attia et al. [2] reported that, in growing rabbits, supplementation with BP doses lower than 350 mg/kg body weight did not improve productive performance. In contrast, Zeedan et al. [9] reported a lower mortality rate and higher hot carcass yield in rabbits supplemented with BP doses greater than 350 mg/kg BW.

On the other hand, PRO is a complex mixture of resinous, gummy, and balsamic substances collected by bees from plant buds, flowers, and exudates [12,13,14,15]. It has been reported that PRO contains more than 180 volatile compounds, mainly polyphenols [16]. The primary polyphenols in PRO are flavonoids and phenolic acids [17], which have antioxidant, immunostimulant, and antimicrobial properties [16,18]. It has been reported that PRO supplementation improves cecum morphology and reduces the presence of *Escherichia coli* and *Salmonella* spp. in the cecum of growing rabbits [13]. In addition, when PRO is added to rabbit diets, serum immunoglobulin concentration is increased, and the antioxidant status of the animals is improved [18]. Likewise, it has been reported that PRO supplementation in rabbits has no toxic effects and helps to reduce the severity of clinical signs and mortality caused by *Pasteurella multocida* [19]. The effects of PRO supplementation in rabbits have been investigated by supplying PRO in capsules [1,2] or by mixing PRO into the basal diet [13,18]. However, to our knowledge, there is no previously published information on the effects of PRO supplementation in drinking water for growing rabbits. Although the most feasible method of administering an additive to rabbits is through feed, it has been reported that PRO supplementation through the basal diet generally has no positive effect on rabbit performance [13,18]. Furthermore, the information available in the literature on PRO supplementation in rabbits remains limited, especially regarding the effects of PRO on meat quality and parasite load in the animals.

Due to the positive effects of BP and PRO, this work hypothesizes that supplementation with BP and PRO will benefit the productive performance of rabbits without affecting health and meat quality. Furthermore, the combination of BP and PRO could act synergistically due to their chemical composition and mechanisms of action. Therefore, the objective of this study was to evaluate the effects of supplementation with bee pollen, propolis, and the combination of both products on productive performance, *Eimeria* oocyst count per gram of feces, blood biometry, blood biochemistry, and meat quality in growing rabbits.

## 2. Materials and Methods

### 2.1. Experimental Location

The experiment was conducted during April and May 2022 at the “Conejos” Centro de Investigación Científica del Estado de México A.C. (COCICEMAC). This site is located in San Miguel Coatlinchán, State of Mexico, Mexico (latitude 19°26′56″ N and longitude 98°52′20″ W). San Miguel Coatlinchán is 2240 m above sea level, the mean annual temperature is 15 °C, and the mean annual precipitation is 645 mm [20]. The Research Ethics and Bioethics Committee approved the experimental procedures for handling the rabbits used in the study of the Universidad Autónoma del Estado de México (Protocol #24-03-2022).

### 2.2. Collection of Bee Pollen and Propolis

BP was collected in hives located in the municipality of Contla de Juan Cuamatzi, State of Tlaxcala, Mexico, located at 2320 masl (latitude 19°20’00″ N and longitude 98°10’00″ W). The BP collection was carried out from September to November 2021 using intermediate pollen traps placed in the hives’ inner part [21]. For proper preservation, the BP was dried at 40 °C and stored in amber jars at room temperature [22].

The PRO was collected in the village Charco de Pantoja, in the municipality of Valle de Santiago, Guanajuato State, Mexico, located at 1709 masl (latitude 20°23’34″ N and longitude 101°11’29″ W). PRO collection was carried out from October 2020 to February 2021 using the plastic net mesh technique [23]. Subsequently, for proper preservation, PRO was cleaned, crushed, and stored in amber jars under refrigerated conditions at −20 °C until further processing [24].

### 2.3. Chemical Composition of Bee Pollen and Propolis, and Preparation and Analysis of Propolis Extract

BP and PRO samples were analyzed in the laboratory to determine the content of dry matter (method 967.03), crude protein (method 981.10), ether extract (method 920.29), and ash (method 942.05), following the procedures previously described by AOAC [25]. The nutritional composition of the BP was: 91.82% dry matter, 20.14% crude protein, 3.95% ether extract, and 2.93% ash. On the other hand, the nutritional composition of the PRO was 90.26% dry matter, 2.55% crude protein, 9.31% ether extract, and 0.85% ash. Therefore, the methodology previously described by Cevk et al. [26] was followed to prepare the PRO extract. Briefly, 100 g of frozen crude PRO was ground using a 1 mm sieve in a Wiley mill (model 4, Arthur Thomas Co., Philadelphia, PA, USA) and mixed with 50 mL of 70% ethanol. Then, the mixture of PRO and ethanol was placed in a hermetically sealed glass container for 48 h (under stirring every 12 h) at room temperature (25 °C). The solution was filtered through Whatman filter paper no. 4 to remove impurities. Finally, the resulting PRO extract solution was stored at −20 °C until further analysis and use (when it was used as a supplement for rabbits) [27].

The total phenolic compound content of the PRO extract was determined using the colorimetric method described by Folin–Ciocalteu [28]. For this, gallic acid was used as a standard, and the absorbance was measured at 765 nm using a UV-VIS spectrophotometer (Aquamate Plus UV-Vis model, Thermo Fisher Scientific^TM^, Waltham, MA, USA). Similarly, the total flavonoid content of the PRO extract was obtained following the colorimetric method using aluminum chloride, as previously described by Chang et al. [29]. In this case, quercetin was used as a standard, and the absorbance was measured at 415 nm using a UV-VIS spectrophotometer (Aquamate Plus UV-Vis, Thermo Fisher Scientific^TM^, Waltham, MA, USA). The total content of phenolic compounds of the PRO was 23.67 mg gallic acid equivalent per g dry weight of the extract. Likewise, the total flavonoid content of the PRO was 9.21 mg quercetin equivalent per g dry weight of the extract.

### 2.4. Animals, Experimental Design, Management, and Diet Composition

All rabbits used in the present study were clinically healthy at the start of the experiment. One hundred sixty freshly weaned California × New Zealand hybrid rabbits (80 males and 80 females), 30 days of age and with an average body weight (BW) of 643 ± 8.0 g, were used. The rabbits were weighed to form four homogeneous groups and were entirely randomized for one of four treatments. Each treatment had 10 replicates (cages), and in each replicate there were four rabbits with similar initial weights and of both sexes (two males and two females). The treatments used were as follows: (1) rabbits fed a basal diet and without BP or PRO in the drinking water (CON); (2) BP500: CON + BP (500 mg/kg BW); (3) PRO50: CON + PRO (50 µL/kg BW); and (4) BP + PRO: CON + BP (500 mg/kg BW) + PRO (50 µL/kg BW). The BP dose (500 mg/kg BW) was chosen based on a previous report in which supplementation with this dose improved the digestibility of ingested nutrients in growing rabbits [9]. The PRO dose (50 µL/kg BW) was chosen based on a previously published study [19] in which the investigators reported that supplementation with this dose of PRO improved immune response and reduced mortality in rabbits.

Treatments were administered five times per week (Monday through Friday) throughout the experimental period. Based on a previous experiment carried out by our work team (unpublished data), we verified that the doses of BP and PRO were well dissolved in the drinking water and that the rabbits consumed this water adequately. Therefore, the BP500, PRO50, or BP + PRO treatments were first applied to 200 mL of drinking water in each of the cages using automatic drinkers, as other authors previously used [30,31,32,33] to supply additives in the drinking water of growing rabbits. After the rabbits in each cage consumed the water supplied with BP500, PRO50, or BP + PRO, the animals had *ad libitum* access to drinking water without treatment. The animals of the CON treatment had the same conditions as the experimental groups but without applying BP or PRO to the drinking water. Instead, drinking water was provided through nipple drinkers. The BP500, PRO50, or BP + PRO treatments were adjusted each week based on the body weights obtained. Oral administration of the treatments was performed in the morning (09:00 h).

Environmental, hygienic, and handling conditions were the same for all rabbits. Also, the health status of the rabbits was monitored throughout the experimental phase. The rabbits were housed in a module with natural ventilation system and thermal insulation on the walls but not on the ceiling. The rabbits were kept in galvanized metal cages 78 cm long, 56 cm wide, and 30 cm high (four rabbits/cage) arranged in a flat-deck system at 45 cm above the floor level. Each cage had a galvanized English-type hopper feeder (15 cm long, 10 cm wide, and 22 cm high) with a capacity of 1.5 kg and a nipple-type automatic drinker. During the entire experimental period (April and May), the average daylight was 13 h.

**Figure 1:**
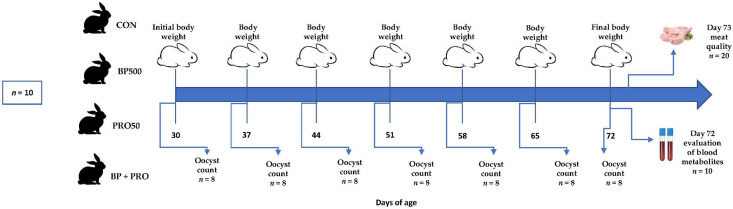
Diagram of the experimental design showing the samples taken during the experiment. CON: basal diet without supplementation with bee pollen (BP) or propolis (PRO); BP500: CON + BP (500 mg/kg BW); PRO50: CON + PRO (50 µL/kg BW); and BP + PRO: CON + BP (500 mg/kg BW) + PRO (50 µL/kg BW).

All rabbits were fed a commercial pelleted basal diet (diameter: 3.5 mm; length: 10 mm) for growing rabbits (Conejo plus, Unión Tepexpan^®^, Texcoco, Mexico), which came from the same production lot. According to the labeled feed package, antibiotics are not included, but 1 g of Diclazuril per ton as coccidiostat is added to prevent *Eimeria* spp. Each week, feed samples were taken and analyzed to determine the chemical composition of the feed following the procedures described by AOAC [25]. The nutritional composition of the basal diet was: 91.71% dry matter, 17.61% crude protein, 5.14% ether extract, 7.48% ash, 32.69% neutral detergent fiber, and 14.81% acid detergent fiber. Water and feed were available *ad libitum* during the 42 days of the experimental phase. Figure 1 shows the experimental design used in this study and the samples taken during the experimental period.

### 2.5. Meteorological Variables and Temperature Humidity Index (THI) Estimation

Ambient temperature and relative humidity were recorded daily using a Traceable^®^ brand hygro-thermometer (model 4040CC, Control Company, Webster, TX, USA) located inside the rabbit facility. With the temperature and relative humidity data, the temperature and humidity index (THI) was estimated using the following equation [34]: THI = T − [(0.31 − 0.31(RH/100)] × [(T − 14.4)], where T is the dry bulb temperature in degrees Celsius (°C), and RH is the percent relative humidity. As a result, the mean values of ambient temperature, relative humidity, and temperature and humidity index (THI) registered during the experimental period were 30.56 ± 0.59 °C, 22.02 ± 0.51%, and 26.62 ± 0.51, respectively.

### 2.6. Growth Performance and Oocyst Count

The body weight (BW) of the rabbits was recorded before morning feeding at the beginning (day 0) of the experimental phase, using a TORREY^®^ brand digital scale (model PCR-40, TORREY Electronics Inc, Houston, TX, USA), with an accuracy of ± 0.5 g. Subsequently, the rabbits were weighed weekly on days 7, 14, 21, 28, 35, and 42 of the experimental periods. The feed intake of the rabbits was recorded each week and divided by the number of days of the period to estimate daily feed intake (DFI, g/d). Likewise, average daily gain (ADG, g/d) was calculated using the weekly BW data. In addition, the rabbits’ feed conversion ratio (FCR) was calculated using the equation: FCR = DFI/ADG.

To evaluate the parasite load of *Eimeria* spp. in rabbits, freshly voided feces samples were collected from eight replicates (cages) of each treatment each week (days 0, 7, 14, 21, 28, 35, and 42). The feces were collected at the same time (9:00 h) on each sampling and were used to perform oocyst counts using the McMaster counting technique [35]. The results were calculated as a number of oocysts per gram of feces (OPG) with the formula [36]: OPG = oocyst count × dilution factor × (sample volume/counting chamber volume). Finally, comparing the number of dead animals to those that started the experimental phase, the overall mortality of the rabbits was calculated, as previously reported by Martínez et al. [37].

### 2.7. Blood Metabolites

To determine hematological and biochemical parameters, at the end of the experimental phase (day 42), blood samples were randomly taken from the ear vein of 10 rabbits (five females and five males) from each treatment. Two 2.5 mL blood samples were taken from each rabbit. Tubes with BD Vacutainer^®^ K2 EDTA anticoagulant (Becton, Dickinson and Company, Franklin Lakes, NJ, USA) were used to collect the first sample, which was stored at 4 °C, for subsequent determination of the complete blood count and differential leukocyte count using an EasyVet^®^ hematology analyzer (QS Kontrolab, Hamburg, Germany), as previously reported by several authors [38,39,40]. BD Vacutainer^®^ (Becton, Dickinson and Company, Franklin Lakes, NJ, USA) anticoagulant-free tubes were used to collect the second blood sample. These blood samples were centrifuged at 3500 rpm for 20 min to obtain blood serum using a refrigerated centrifuge (Sigma 2–16 k, Sigma Laborzentrifugen GmbH, Osterode am Harz, Germany). The blood serum was stored in Eppendorf tubes and frozen at −20 °C. Finally, for the analysis of the blood serum samples, an EasyVet^®^ autoanalyzer (QS Kontrolab, Hamburg, Germany) and Spinreact kits (Barcelona, Spain) were used to determine the contents of glucose (kit 41011), cholesterol (kit 41021), albumin (kit 1001020), globulin (kit 1001032), total protein (kit 1001291), urea (kit 41041), uric acid (kit 41001), bilirubin (kit 1001046), creatinine (kit 1001113), liver enzymes (alkaline phosphatase (kit MX41233), lactate dehydrogenase (kit MX41274), and aspartate aminotransferase (kit MX41264)), calcium (kit 1001060), and phosphorus (kit 1001155), as described by other authors [38,41].

### 2.8. Carcass Yield and Meat Quality

After obtaining the final body weight (day 42 of the experiment), all rabbits were kept fasting for 12 h. Subsequently, 20 rabbits (ten males and ten females) from each treatment were sacrificed following the procedures of the Mexican Official Standard (NOM-033 SAG/ZOO-2014). After completing the slaughter and bleeding of the rabbits, all internal organs, skin, feet, and heads were separated from the carcass, and the hot carcass weight (HCW) was recorded. The hot carcass yield (HCY) was estimated by the equation: HCY = (HCW / final BW) × 100. Subsequently, the leg muscles (HLM) and *Longissimus dorsi* muscles (LDM) were removed from the hot carcass, following procedures similar to those described and recommended by Blasco and Ouhayoun [42]. Finally, HLM and LDM muscles were stored in a freezer at −20 °C until further analysis.

Before meat quality analyses, samples were thawed at 4 °C for 24 h. Then, meat pH was determined using the procedures previously described by Orzuna-Orzuna et al. [43]. For this, 3 g of muscle was weighed in triplicate from the HLM sample of each rabbit. Subsequently, using a Waring 51BL32 blender (model 700, Torrington, CT, USA), each portion of muscle was homogenized with 20 mL of deionized water. Finally, each sample was measured in triplicate for pH with a Hanna^®^ brand pH meter (Model HI 98127, Waterproof Tester, Woonsocket, RI, USA).

Meat color parameters were measured in triplicate in LDM samples from each rabbit using the procedures described by Miltenburg [44]. For this, lightness (L*), redness (a*), and yellowness (b*) were measured in triplicate with a Minolta CM-2006d spectrophotometer (Konica model, Minolta Holdings Inc., Osaka, Japan). In addition, cooking loss (CL) was evaluated in LDM samples (one sample per rabbit), as previously reported by Manchetti et al. [45]. Therefore, 2.5 cm thick steaks were grilled on an electric grill (Toastmaster cool-edge grill, Macon, MO, USA). The internal temperature of the meat was monitored with a Taylor^®^ brand thermometer (model 99878, Seattle, WA, USA), and when it reached 70 °C, the steaks were removed from the grill and allowed to cool for 1 h at room temperature (20–25 °C). The percentage of CL was estimated with the equation [46]: CL, % = ((Wr − Wc)/Wr) × 100, where Wr is the raw weight and Wc is the cooked weight of the meat samples used.

HLM samples from each rabbit were separately ground and homogenized for 5 min using a Ship to Shore brand meat grinder (Model 99598, Camarillo, CA, USA) [47]. Subsequently, a FOSS FoodScan™ near-infrared spectrophotometer was used to determine in triplicate the content (g/100) of moisture, protein, fat, and collagen, following the procedures described by Anderson [48].

### 2.9. Statistical Analysis

All data were analyzed by analysis of variance (ANOVA) using SAS statistical software [49]. Before statistical analysis, the normality of the data was evaluated with the Shapiro–Wilk test using the UNIVARIATE procedure. Likewise, the productive performance and OPG data were analyzed using a completely randomized experimental design with repeated measures over time. The MIXED procedure was used, and each cage was considered the experimental unit. Finally, different variance–covariance structures were tested to fit the statistical model, and the compound symmetry structure was chosen for the productive performance and OPG variables because it showed the best fit following the criteria of lowest AIC and BIC values [50]. The structure of the statistical model used was:

Y*_ijk_* = *µ* + T*_i_* + W*_j_* + (T × W)*_ij_* + R*_k_* + e*_ijk_* (1)

In this model, Y*_ijk_* represents the value observed in treatment *i* in week *j* for rabbit *k*; *µ* represents the overall mean; T*_i_* represents the fixed effect of the *i*-th treatment (*i* = 1 (CON), 2 (BP500), 3 (PRO50), 4 (BP + PRO)); W*_j_* represents the fixed effect of the *j*-th week of the experimental period (*j* = 1, 2, …, 6,); (T × W)*_ij_* represents the fixed effect of the interaction between the *j*-th week and the *i*-th treatment; R*_k_* represents the random effect of a cage (repetition) within treatment (*k* = 1, 2, 3, …, 40); and e*_ijk_* represents the random error.

The variables of blood biometry, blood biochemistry, meat quality, and HCY were analyzed using a completely randomized design. For this, the GLM procedure of SAS [44] was used, and each rabbit was considered the experimental unit. The structure of the final statistical model used was as follows:

Y*_ij_* = *µ* + T*_i_* + e*_ij_* (2)

In this model, Y*_ij_* represents the observations, *µ* represents the overall mean, T*_i_* represents the fixed effect of the *i*-th treatment, and e*_ij_* is the random error. For all variables analyzed, treatment means were compared using Tukey’s test. The mortality rate was analyzed as a percentage using chi-square analysis [1]. A statistically significant effect was considered to be present when *p* ≤ 0.05. In addition, *p* > 0.05 and ≤ 0.10 was considered as tending to be significant.

## 3. Results

### 3.1. Growth Performance and Oocyst Count

Table 1 shows that at the end of the experimental phase, rabbits assigned to the PRO50 treatment were 6.1% heavier (*p* < 0.05) than rabbits assigned to the CON treatment. ADG was 8.8% greater (*p* = 0.04) in the PRO50-group rabbits than in the CON-treatment rabbits. FCR was 7.1% lower (*p* = 0.03) in PRO50-treatment rabbits than in CON-treatment rabbits. Compared to the CON treatment, rabbits in the PRO50 and BP + PRO treatments had 50 and 55% lower OPG (*p* < 0.05), respectively. On the other hand, mortality was higher (*p* = 0.05) in the BP500-treatment rabbits than in the rabbits assigned to the other treatments (Table 1).

### 3.2. Hematological Parameters

Higher (*p* = 0.02) percent hematocrit was observed in BP500-supplemented rabbits than in CON rabbits (Table 2). Compared to CON- and BP500-treatment rabbits, rabbits supplemented with PRO50 and BP + PRO had lower blood hemoglobin concentration (*p* = 0.009). Mean corpuscular volume was lower (*p* = 0.02) in PRO50-treatment rabbits than in CON rabbits. Lower (*p* = 0.001) blood concentration of monocytes was observed in BP + PRO-treatment rabbits than in rabbits assigned to the other treatments. Rabbits in the PRO50 treatment had higher (*p* < 0.05) blood concentrations of band neutrophils and eosinophils than in the CON, BP500, and BP + PRO treatments.

### 3.3. Blood Biochemistry

Rabbits supplemented with BP500 had lower (*p* = 0.001) serum urea concentration compared to rabbits in the CON, BP500, and BP + PRO treatments. In addition, rabbits supplemented with BP500 had a higher albumin/globulin ratio (*p* = 0.04) and lower serum total protein and globulin concentration (*p* < 0.05) than CON-treatment rabbits. Serum aspartate aminotransferase concentration tended to be lower in BP500-supplemented rabbits than in CON-treatment rabbits (*p* = 0.09; Table 3).

### 3.4. Carcass Yield and Meat Quality

Higher (*p* = 0.002) HCY was observed in rabbits supplemented with BP500 and PRO50 than in rabbits assigned to the CON and BP + PRO treatments (Table 4). The pH was higher (*p* = 0.001) in meat from rabbits supplemented with BP + PRO than in meat from rabbits in the other treatments. PRO50 supplementation reduced (*p* < 0.05) the CL of meat compared to the other treatments. However, lower fat content (*p* = 0.05) and higher moisture content (*p* = 0.03) were observed in meat from rabbits supplemented with BP500 than in meat from CON rabbits (Table 4).

## 4. Discussion

### 4.1. Growth Performance and Oocyst Count

In some review articles [6,51], it has been mentioned that dietary supplementation with BP or PRO may improve the taste of the feed offered, which could result in higher DFI. However, in the present study, supplementing with an aqueous solution of BP, PRO, and BP + PRO did not affect DFI. Similar responses were previously reported by Piza et al. [52] in rabbits supplemented with increasing doses of PRO (0, 500, 1000, and 1500 mg/kg DM); and by El-Hammady et al. [53] in adult rabbits supplemented with 500 or 1000 mg/d of BP. Moreover, Attia et al. [1,10] did not observe significant changes in the DFI of growing rabbits supplemented with capsules containing increasing doses (150, 200, and 300 mg/kg BW) of BP, PRO, or the combination of both products. These results suggest that supplementation with BP, PRO, or BP + PRO does not affect DFI, regardless of the dose and route of administration used.

Zeedan et al. [9] observed that in rabbits, supplementation with BP increases the production of volatile fatty acids in the cecum and the activity of amylase, lipase, and protease in the intestinal contents. In addition, BP has been reported to increase intestinal villus length by more than 50% [10]. Consequently, rabbits supplemented with BP would be expected to have higher ADG and final BW; however, in the present study, ADG and final BW were unaffected by supplementation with either BP500 or BP + PRO. On the other hand, in the present study, higher ADG and final BW were observed in rabbits on PRO50 treatment. This result could be related to the lower *Eimeria* OPG count observed in rabbits supplemented with PRO50 because there is a strong negative correlation (r = −0.91) between BW and *Eimeria* spp. OPG count [54]. Furthermore, these results suggest that PRO supplementation could replace some antibiotics (e.g., zinc bacitracin) commonly used as growth promoters in rabbits [10]. Previous studies [13,18] have reported that, in growing rabbits, dietary supplementation with PRO (150, 250, 300, and 500 mg/kg DM) improves the total antioxidant capacity and increases between 8 and 22% the serum concentration of immunoglobulins (IgM, IgY, and IgG). These effects could improve the health of rabbits and result in higher ADG and final BW. Furthermore, North et al. [55] reported that, in the cecum of rabbits, supplementation with quercetin (a typical flavonoid of propolis) increases the relative abundance of microorganisms (*Eubacteriaceae, Peptococcaceae,* and *Natranaerobiaceae*) that are positively correlated with ADG. Likewise, the addition of quercetin in diets for rabbits increases up to 7.6% the serum concentration of growth hormone [56]. Similar effects of the consumption of PRO and its flavonoids in the present study partially explain the observed increases in ADG and final BW.

FCR was not affected by supplementation with BP500 and BP + PR, suggesting that the doses of BP and BP + PR used in the present study do not improve feed efficiency in growing rabbits. In similar studies, Attia et al. [1,10] also did not observe significant changes in the FCR of growing rabbits supplemented with various doses of BP or BP + PRO.

On the other hand, FCR was lower in rabbits supplemented with PRO50. PRO supplementation has been reported to improve rabbits’ digestibility of organic matter and ingested crude protein [57]. North et al. [55] observed that flavonoid (quercetin) supplementation increases the relative abundance of microbial families (*Erysipelotrichaceae* and *Haloplasmataceae*) that are negatively correlated with FCR. Consequently, similar PRO and flavonoid consumption effects observed in our study could partially explain the lower FCR in rabbits on PRO50 treatment. The lower FCR observed in rabbits supplemented with PRO50 suggests that PRO could improve the profitability of rabbit production systems since FCR is a crucial indicator for judging an animal production system [11].

Coccidiosis is an infection caused by *Eimeria* protozoa, which in rabbits causes growth retardation and high mortality [58]. In the present study, the OPG count of *Eimeria* decreased in rabbits supplemented with PRO50 and BP + PRO. This result suggests that these products could be used as natural coccidiostats for growing rabbits. This hypothesis is supported by the average OPG values observed in rabbits supplemented with PRO50 and BP + PRO, which were lower than the range (4000–5000 OPG) at which the application of prophylactic treatment against *Eimeria* protozoa is required [59].

On the other hand, although mortality was higher in rabbits supplemented with BP500, the mortality observed in the present study was high (between 17.5 and 40%) for all treatments. In the present study, a seasonal effect and climatic conditions might have an essential role in the high mortality rates observed [60], particularly if considering that the average ambient temperature during the experimental period was high (30.6 ± 0.59 °C).

### 4.2. Hematological Parameters and Blood Biochemistry

When evaluating a new feed additive for domestic animals, it is essential to analyze the effects of the consumption of the additive on animal health [31]. According to Diaz Cano et al. [61], knowledge of reference values for blood metabolites in rabbits provides valuable information on the health status of the animals. In particular, hematological parameters provide information on rabbits’ visceral organ infections, inflammation, and necrosis [62]. Except for leukocytes, monocytes, and eosinophils, in the present study the hematological parameters of rabbits from all treatments were within the normal range reported in the literature for healthy rabbits [63,64,65]. These results suggest that supplementation with BP500, PRO50, and BP + PRO does not affect the hematological system of growing rabbits.

The blood concentration of leukocytes was similar between treatments. However, the blood concentration of leukocytes in CON treatment rabbits was higher than the normal range reported for healthy rabbits [63]. In addition, compared with BP + PRO treatment rabbits, the percentage of monocytes in CON treatment rabbits was significantly higher and above the normal range (3.17–4.67 × 10^3^/mL) [63]. These results could be associated with the higher OPG observed in CON treatment rabbits because the blood concentration of leukocytes and monocytes increases in rabbits infected with *Eimeria* parasites [66]. Furthermore, it has been reported that the blood concentration of leukocytes increases in animals with intoxication (endogenous or exogenous) or infectious diseases [67]. On the other hand, the blood concentration of monocytes increases in rabbits with chronic infection and inflammation [66]. Consequently, the results observed in the present study for leukocytes and monocytes suggest that supplementation with BP500, PRO50, or BP + PRO did not negatively affect the health of growing rabbits.

Serum glucose concentration and lipid metabolites are indicators of rabbits’ energy status [68]. In the present study, serum glucose and cholesterol concentrations were similar between treatments and were within the range reported in the literature for healthy rabbits [62]. This result suggests that supplementation with BP500, PRO50, and BP + PRO did not affect the energy status of growing rabbits.

Supplementation with BP500 reduced serum globulin and total protein levels. However, rabbits from all treatments had serum urea, albumin, globulin, and total protein concentrations within the reference range considered normal for healthy rabbits [62]. This result suggests that the doses used of BP, PRO, and BP + PRO have no negative effects on protein catabolism and no adverse effects on the nutritional status of growing rabbits.

If analyzed at appropriate reference intervals, serum uric acid and creatinine concentrations can be used as biomarkers of renal function status in rabbits [61]. For example, serum creatinine levels increase above the reference interval when animals have chronic and acute renal failure [69]. In the present study, serum creatinine and uric acid concentrations were similar between treatments, and serum levels of these metabolites were within the range reported in the literature for healthy rabbits [65]. These results suggest that supplementation with BP or PRO does not affect the renal health of rabbits.

Serum levels of hepatic enzymes are often used as markers of liver disease [70]. In addition, alkaline phosphatase is associated with other disorders, such as intestinal and generalized tissue damage [71]. In the present study, rabbits from all treatments had serum levels of alkaline phosphatase, lactate dehydrogenase, and aspartate aminotransferase higher than the normal range reported for healthy rabbits [62,64]; however, there were no differences between treatments. This result suggests that supplementation with BP or PRO does not affect growing rabbits’ hepatic or intestinal health.

It has been mentioned that serum calcium and phosphorus levels can be used as good indicators of nutritional status in domestic animals because their variability is low [72,73]. In the present study, serum calcium and phosphorus concentrations were similar between treatments. In addition, rabbits from all treatments had serum values for calcium and phosphorus within the range reported for healthy rabbits [63]. This result suggests that supplementation with BP or PRO does not affect growing rabbits’ mineral and nutritional status.

### 4.3. Carcass Yield and Meat Quality

In the present study, supplementation with BP500 and PRO50 increased HCY. In a similar study, Zeedan et al. [9] also observed increased HCY in rabbits supplemented with increasing doses of BP (0, 200, 500, and 700 mg/kg BW for 70 days). Similarly, Waly et al. [58] reported higher HCY in growing rabbits supplemented with increasing doses of PRO (0, 100, 150, and 200 mg/kg DM for eight weeks).

The pH is considered one of the most critical parameters determining rabbit meat quality because it is related to its color, flavor, water-holding capacity (WHC), tenderness, and shelf life [74]. For example, when the pH of meat is higher than 6, the development of proteolytic microorganisms increases [75]. In the present study, only supplementation with BP + PRO increased meat pH. This result suggests that when administered individually, BP and PRO do not significantly alter the physicochemical properties of meat. However, simultaneous administration of BP and PRO could reduce rabbit meat’s quality and shelf life.

Previous studies have reported that meat color can be modified by changes in fat content and pH [76,77]. In the present study, lower fat content and higher pH were observed in meat from rabbits supplemented with BP500 and BP + PRO, respectively. However, meat colors (L*, a*, and b*) were similar among rabbits of all treatments, indicating that supplementation with BP or PRO does not affect the appearance of rabbit meat. Based on the literature consulted, there is no previously published information on the effects of supplementation with BP, PRO, or the combination of BP and PRO on rabbit meat color. However, Prakatur et al. [78] also did not observe significant changes in L*, a*, and b* of broiler meat supplemented with increasing doses (0, 200, 250, 500, 1000, and 2000 mg/kg DM for 42 days) of BP, PRO, or the combination of BP and PRO.

In the present study, PRO50 supplementation reduced the CL of meat. This result suggests that PRO supplementation can improve the WHC of rabbit meat because CL and WHC are negatively correlated [76]. Limited information on the effects of BP and PRO supplementation on the CL of rabbit meat makes it difficult to explain the results observed in the present study. However, it has been reported that CL increases when there is oxidative damage to the meat [79]. The PRO used in the present study contained flavonoids, which improve the oxidative stability of meat [80,81]. This effect partially explains the lower CL observed in rabbits supplemented with PRO50.

The chemical composition of rabbit meat from all treatments had values within the normal range [82]. This result suggests that supplementation with BP500, PRO50, or BP + PRO did not affect the nutritional quality of rabbit meat.

## 5. Conclusions

The results of this study indicate that propolis supplementation (50 µL/kg BW) can be used to prevent coccidiosis and as a natural growth promoter in rabbits without affecting animal health and meat quality. However, more research is needed to understand and explain the impact of bee pollen and propolis supplementation on productivity, parasite load, health status, and the meat quality of growing rabbits.

## Figures and Tables

**Table 1:** Growth performance and mortality of rabbits supplemented with bee pollen and propolis in a trial from 30 to 72 days of age.

Parameters	Treatments				SEM	*p*-Value		
	CON	BP500	PRO50	BP + PRO		Treatment	Week	Treatment × Week
Cages (*n*)	10	10	10	10				
Rabbits (*n*)	40	40	40	40				
Initial body weight, g	639	633	642	641	23.21	0.20	-	-
Final body weight, g	2168 ^b^	2197 ^ab^	2306 ^a^	2180 ^ab^	66.31	0.03	<0.0001	0.12
Average daily gain (ADG), g/d	36.4 ^b^	37.3 ^ab^	39.6 ^a^	36.6 ^ab^	1.52	0.04	<0.0001	0.11
Daily feed intake (DFI), g/d	107.6	112.2	108.9	104.9	3.75	0.06	<0.0001	0.80
Feed conversion ratio (FCR), DFI/ADG	2.95 ^a^	3.00 ^ab^	2.74 ^b^	2.86 ^ab^	0.28	0.03	<0.0001	0.28
Initial oocyst/g feces (OPG)	7.1	0.0	0.0	0.0	3.37	0.14	-	-
Oocyst/g feces (OPG)	6159.8 ^a^	5546.0 ^ab^	3037.5 ^bc^	2770.8 ^c^	1004.15	0.02	0.07	0.08
Mortality, %	22.5 ^b^	40.0 ^a^	22.5 ^b^	17.5 ^b^	8.16	0.05	-	-

CON: basal diet without supplementation with bee pollen (BP) or propolis (PRO); BP500: CON + BP (500 mg/kg BW); PRO50: CON + PRO (50 µL/kg BW); and BP + PRO: CON + BP (500 mg/kg BW) + PRO (50 µL/kg BW). SEM—standard error of the treatment means; ^a,b,c^—means within a row with different subscripts differ when *p* ≤ 0.05.

**Table 2:** Hematological profile of rabbits supplemented with bee pollen and propolis in a trial from 30 to 72 days of age.

Parameter	Treatment				SEM	*p*-Value
	CON	BP500	PRO50	BP + PRO		
Rabbits (*n*)	10	10	10	10		
Hematocrit, %	37.20 ^b^	38.79 ^a^	37.14 ^b^	37.04 ^b^	0.509	0.02
Hemoglobin, g/dL	12.84 ^a^	12.92 ^a^	12.29 ^b^	12.23 ^b^	0.177	0.009
Red blood cells, 10^6^/mL	6.52 ^ab^	6.77 ^a^	6.38 ^ab^	6.29 ^b^	0.141	0.02
Mean corpuscular volume, fL	60.12 ^a^	59.00 ^ab^	58.45 ^b^	60.40 ^a^	0.560	0.02
Mean corpuscular hemoglobin, pg	19.68	19.65	19.12	19.42	0.222	0.08
Mean corpuscular hemoglobin concentration, g/dL	33.01	33.19	33.22	33.86	0.553	0.28
Platelets, 10^3^/mL	256.60	253.40	247.70	247.40	8.083	0.42
Leukocytes, 10^3^/mL	14.63	7.68	7.70	7.45	3.477	0.15
Lymphocytes, 10^3^/mL	12.80	14.10	12.50	12.90	1.236	0.36
Monocytes, 10^3^/mL	10.00 ^a^	8.40 ^a^	9.70 ^a^	6.10 ^b^	0.812	0.001
Segmented neutrophils, 10^3^/mL	75.90	76.80	73.50	73.40	1.799	0.09
Band neutrophils, 10^3^/mL	0.40 ^b^	0.40 ^b^	1.90 ^a^	1.10 ^b^	0.374	0.02
Eosinophils, 10^3^/mL	1.00 ^c^	3.00 ^b^	4.80 ^a^	2.10 ^bc^	0.593	<0.0001
Basophils, 10^3^/mL	0	0	0	0	0	0
Plasma protein, g/dL	7.82	7.81	7.62	7.66	0.164	0.39

CON: basal diet without supplementation with bee pollen (BP) or propolis (PRO); BP500: CON + BP (500 mg/kg BW); PRO50: CON + PRO (50 µL/kg BW); and BP + PRO: CON + BP (500 mg/kg BW) + PRO (50 µL/kg BW). SEM—standard error of the treatment means; ^a,b,c^—means within a row with different subscripts differ when *p* ≤ 0.05.

**Table 3:** Blood serum biochemistry of rabbits supplemented with bee pollen and propolis in a trial from 30 to 72 days of age.

Parameter	Treatment				SEM	*p*-Value
	CON	BP500	PRO50	BP + PRO		
Rabbits (*n*)	10	10	10	10		
Glucose, mg/dL	119.20	111.80	119.10	116.90	4.805	0.28
Urea, mg/dL	39.50 ^a^	32.60 ^b^	38.20 ^a^	37.50 ^a^	1.423	0.001
Cholesterol, mg/dL	63.30	60.30	66.10	62.50	5.326	0.44
Total protein, g/dL	7.94 ^a^	7.09 ^b^	7.42 ^ab^	7.52 ^ab^	0.258	0.02
Albumin, g/dL	3.23 ^ab^	3.18 ^b^	3.30 ^a^	3.25 ^ab^	0.041	0.05
Globulin, g/dL	4.70 ^a^	3.91 ^b^	4.12 ^ab^	4.23 ^ab^	0.242	0.03
Albumin/globulin	0.73 ^b^	0.81 ^a^	0.79 ^ab^	0.76 ^ab^	0.026	0.04
Bilirubin, mg/dL	0.33	0.30	0.38	0.28	0.051	0.13
Uric acid, mg/dL	0.58	0.30	0.57	0.46	0.116	0.09
Creatinine, mg/dL	1.09	1.01	1.21	1.20	0.080	0.08
Alkaline phosphatase, UI/dL	297.30	349.00	313.80	363.40	25.30	0.07
Lactate dehydrogenase, UI/dL	472.90	334.40	526.80	499.10	68.39	0.06
Aspartate aminotransferase, UI/dL	62.70	47.10	64.10	48.20	8.63	0.09
Calcium, mg/dL	14.35	14.29	14.58	14.27	0.30	0.46
Phosphorus, mg/dL	5.84	5.94	6.32	5.81	0.23	0.12

CON: basal diet without supplementation with bee pollen (BP) or propolis (PRO); BP500: CON + BP (500 mg/kg BW); PRO50: CON + PRO (50 µL/kg BW); and BP + PRO: CON + BP (500 mg/kg BW) + PRO (50 µL/kg BW). SEM—standard error of the treatment means; ^a,b^—means within a row with different subscripts differ when *p* ≤ 0.05.

**Table 4:** Carcass yield and meat quality of rabbits supplemented with bee pollen and propolis for 42 days and slaughtered at 72 days of age.

Parameter		Treatment				SEM	*p*-Value
		CON	BP500	PRO50	BP + PRO		
Rabbits (*n*)	Muscle used	20	20	20	20		
Hot carcass yield (HCY), %		54.78 ^b^	57.41 ^a^	57.59 ^a^	55.04 ^b^	0.91	0.002
Meat pH	Hind leg muscle	5.96 ^b^	5.99 ^b^	5.98 ^b^	6.04 ^a^	0.02	0.001
Lightness (L*)	*Longissimus dorsi* muscle	53.49	52.67	54.08	52.44	0.86	0.11
Redness, (a*)	*Longissimus dorsi* muscle	1.90	1.82	2.01	1.69	0.22	0.18
Yellowness, (b*)	*Longissimus dorsi* muscle	7.18	7.09	7.39	7.34	0.18	0.11
Cooking loss (CL), %	*Longissimus dorsi* muscle	33.24 ^a^	35.31 ^a^	28.97 ^b^	37.05 ^a^	1.92	0.0006
Protein, g 100 g^−1^	Hind leg muscle	21.42	21.70	21.38	21.53	0.17	0.25
Fat, g 100 g^−1^	Hind leg muscle	2.98 ^a^	1.93 ^b^	2.70 ^ab^	2.62 ^ab^	0.39	0.05
Moisture, g 100 g^−1^	Hind leg muscle	74.63 ^b^	75.38 ^a^	74.89 ^ab^	74.85 ^ab^	0.35	0.03
Collagen, g 100 g^−1^	Hind leg muscle	0.97	0.99	1.03	1.00	0.02	0.17

CON: basal diet without supplementation with bee pollen (BP) or propolis (PRO); BP500: CON + BP (500 mg/kg BW); PRO50: CON + PRO (50 µL/kg BW); and BP + PRO: CON + BP (500 mg/kg BW) + PRO (50 µL/kg BW). SEM—standard error of the treatment means; ^a,b^—means within a row with different subscripts differ when *p* ≤ 0.05.

## Data Availability

The datasets used and analyzed during the current study are available from the corresponding author on reasonable request.
